# Illustrative presentations of the failing heart in the acutely ill child: two case reports

**DOI:** 10.1186/1757-1626-2-9326

**Published:** 2009-12-15

**Authors:** Derya Caglar, Julie C Brown, Eileen J Klein

**Affiliations:** 1 Department of Pediatrics, University of Washington School of Medicine, and Seattle Children's Hospital, 4800 Sand Point Way NE, Mailstop B-5520, Seattle, WA, 98105, USA

## Abstract

Two cases of pediatric patients with heart failure are presented. One child presented with vomiting and the other a child with a history of asthma who had respiratory distress. Though their presenting complaints are common, the diagnosis was made based on careful examination and consideration of abnormal findings. Abnormal vital signs (tachycardia, bradycardia, hypotension) or physical exam findings (heart murmur or gallop, right upper quadrant pain) can provide important clues to accurate and timely diagnosis.

## Introduction

Diagnosing the rare patient with myocarditis or cardiomyopathy in a child in the acute care setting is essential, but frequently such patients present with symptoms more commonly associated with other illness or disease. How can the practitioner identify the clues that the common symptom presentation is anything but common? We present two cases of children with heart failure and discuss the important aspects of diagnosis.

## Case presentation

### Case report 1

A previously healthy 3-year-old Hispanic female is brought to the emergency department (ED) for vomiting. Her parents reported that she had three episodes of non-bloody, non-bilious emesis over 1 day and seemed fatigued. They reported no fevers, diarrhea, abdominal pain, rashes, or upper respiratory symptoms. Her past medical history was unremarkable except for an episode of hemolytic anemia at 15 months following a viral illness. The patient was initially taken to her primary care provider where she was found to be less interactive than usual, pale, and unwilling to walk or play.

The patient was sent to the ED for further evaluation. She was somnolent but responsive to tactile stimulation. Her extremities were cool and mottled with delayed capillary refill. The lungs were clear and the heart tones were normal with no murmurs, gallops, or rubs. Her heart rate was 40 beats per minute. Electrocardiogram (ECG) monitoring revealed intermittent third degree heart block with bradycardic episodes, during which she had decreased responsiveness. A venous blood gas revealed metabolic acidosis, with a pH 7.09 and a base deficit of 16. She was given a 10 mg/kg normal saline bolus, started on an isoproterenol drip, and placed on 6 liters of oxygen by mask. Her heart rate increased to 140 and her oxygen saturation was maintained at 100%. A chest radiograph showed no cardiomegaly, effusion, pulmonary infiltrate, or edema. She was subsequently transferred to the pediatric intensive care unit for ongoing evaluation and treatment.

Laboratory values indicated myocardial damage: BNP 1150, troponin I 50.54, and CK-MB 65. An echocardiogram revealed normal ventricular contractility with left ventricular wall edema and an abnormal shortening fraction of 29%. Multiple studies were sent, including blood cultures, viral PCR for enterovirus, Mycoplasma, EBV, CMV, parvovirus, and Lyme titers, all of which returned negative. Rheumatologic studies were sent and were significant for low complement levels.

A cardiac catheterization and biopsy showed severe lymphocytic infiltration with myocyte necrosis, myocardial edema, and early interstitial collagen deposition. She was treated with IVIG and methylprednisone for presumed myocarditis with improvement of her symptoms, lab values, and rhythm.

The patient was discharged home on hospital day 12 on captopril and a prednisone taper with a return to normal cardiac function. Follow-up in cardiology clinic revealed a normal exam, return to baseline behavior, and normal echocardiogram.

### Case report 2

An 8-year-old African-American male with a history of asthma presented to his primary care provider for increasing exercise intolerance, shortness of breath, and cough for one day. He was started on methylprednisone and albuterol for possible exacerbation of his asthma. He returned to his pediatrician four days later for follow-up with increasing respiratory distress and shortness of breath and was referred to the ED for further evaluation.

On arrival to the emergency department, the patient was alert, awake, and cooperative. He walked into the room effortlessly, though did appear to have mild shortness of breath. His examination was notable for quiet heart sounds a possible holosystolic murmur and a quiet gallop. Although his liver edge was hard to feel, given the size of the patient, he was tender in the right upper quadrant. His extremities were warm and well-perfused, although radial pulses felt weak. He was placed on oxygen via nasal cannula. A chest radiograph showed an enlarged cardiac silhouette with pulmonary edema. An echocardiogram demonstrated a severely dilated left ventricle, severely depressed left ventricular function, moderate mitral and tricuspid regurgitation, and elevated right-sided pressures. He was noted to have a shortening fraction of 14%. Initial labs were unremarkable except a BNP 1150. He was admitted to the cardiac intensive care unit for further evaluation and treatment.

The patient was initially maintained on oxygen via nasal cannula but developed increased work of breathing that required CPAP and diuretic therapy. He was placed on milrinone captopril, and furosemide to improve cardiac function and decrease fluid overload. A cardiac catheterization confirmed a dilated cardiomyopathy and biopsy showed nonspecific acute inflammation. Prophylactic heparin anticoagulation was initiated due to his dilated ventricles and poor ventricular function. Viral studies and blood cultures were negative except for one culture that grew *Streptococcus viridans*. He was placed on ceftriaxone for possible bacteremia.

Despite slow improvement in cardiac enzyme studies, the patient continued to require increasing amounts of milrinone and diuretic therapy. He developed increasing respiratory distress with increasing CPAP needs and sildenafil was initiated due to concern for pulmonary hypertension. His condition continued to deteriorate and the patient was placed on a waiting list for cardiac transplantation.

## Discussion

Early and accurate diagnosis of a child with heart failure can be a difficult task. Though the majority of cases of pediatric heart failure occur in the first year of life in children with congenital heart disease, older children may present with failure due to acquired disease such as myocarditis or cardiomyopathy, as illustrated by our cases.

### Pathophysiology

Myocarditis is caused by inflammation of the myocardium with necrosis of the myocytes.

Viral infections, predominantly adenovirus and coxsackievirus, are responsible for the majority of cases. However, bacteria, drugs, and toxic exposures have also been implicated [1-3]. Though an infectious agent often causes the initial damage, molecular mimicry between the inciting agent and myocytes often leads to an autoimmune response that increases inflammatory infiltration of the myocardium and allows disease progression.

As damage to the myocardium continues, patients may develop varying degrees of ventricular dysfunction and/or dilatation. Dilated cardiomyopathy is characterized by a dilated left ventricle with systolic dysfunction and signs of congestive heart failure[4]. Several studies have found an incidence between 0.34 and 1.09 cases per 100,000 patients per year with the majority of identified cases caused by myocarditis [5].

Heart failure occurs when the heart is unable to provide adequate blood volume to meet the circulatory and metabolic demands of the body. In a healthy child, the heart can compensate for increased demands by increasing the heart rate, increasing the contractility of the ventricles, increasing preload, or decreasing peripheral resistance, all of which lead to increased flow of blood volume to those areas in need of more oxygen. When the demands of the body placed on the heart exceed its ability to compensate, failure occurs.

In fulminant cases, patients present in decompensated shock with classic signs of pulmonary edema, tachycardia, and hypoxia making the diagnosis clear and subsequent treatment immediate. But the practitioner must also consider the possibility of cardiac disease in those individuals with more atypical symptoms or insidious onset. Older patients may complain of shortness of breath, fatigue, or wheezing, making the presentation difficult to distinguish from asthma exacerbations [6]. Younger children may present with non-specific malaise or decreased energy, as are often seen with viral illnesses, sometimes associated vomiting is the primary symptom concerning the parents [7].

Patients may present with symptoms of low cardiac output including fatigue, cool extremities, dizziness, altered level of consciousness, or syncope. Initial damage to the myocardium can lead to exercise intolerance from decreased myocardial function. Increasing dilatation of the heart chambers with poor contractility and function can lead to pulmonary congestion. Atrio-ventricular nodal infiltration presents as rhythm disturbances and complete heart block while ventricular dysrhythmias can lead to seizures, chest pain, and sudden death.

### Diagnosis

There are no definitive tests for myocarditis or cardiomyopathy. The clinician must be alert for the possibility of heart failure in any patient, and should start with a thorough physical examination, paying special attention to presenting vital signs.

The patient's heart rate may be a clue of underlying cardiac disease. Tachycardia is commonly seen and usually relates to fever, fear, or a pulmonary problem. However, tachycardia may also be seen in a failing heart with arrhythmias or decrease in ventricular contractility, causing poor cardiac output. Bradycardia is more rarely seen in the acutely ill patient and merits further evaluation for cardiac dysfunction.

Respiratory rate and blood pressure can both help identify patients at high risk for cardiac disease. Though tachypnea is usually seen in patients with fever and pulmonary processes, the practitioner must consider the possibility of pulmonary edema from a failing heart. Hypotension is an ominous sign. While most patients with mild heart failure remain normotensive early in the course of illness, cardiac decompensation with progression of illness will lead to hypotension and cardiogenic shock.

Patients with heart failure may present with a normal cardiovascular examination, but a careful survey can reveal important markers of cardiac disease. A new murmur or gallop may be heard with valvular or myocardial dysfunction. A hyperactive precordium may be felt on palpation as the dysfunctional myocardium struggles to maintain cardiac output. Diminished peripheral pulses or mottled, cool extremities can provide valuable evidence of diminished peripheral perfusion. Poor perfusion may indicate poor ventricular function instead of dehydration, even in light of vomiting or decreased oral intake.

Wheezing is the classic sign of asthma exacerbation but can also be an important physical finding in patients with myocarditis or cardiomyopathy. Children with cardiac dysfunction are most likely to present with increased work of breathing, tachypnea, retractions, and nasal flaring as opposed to rales or other adventitial lung sounds. The practitioner should consider a cardiac etiology for respiratory symptoms in a known asthmatic if conventional treatments fail to produce improvement or there are other clues present (e.g., vital signs, heart exam, peripheral pulses).

Abdominal pain can be a presenting symptom in patients with heart failure. Venous congestion due to a failing heart can not only cause pulmonary edema, but also lead to hepatomegaly. Right upper quadrant pain and hepatomegaly in the presence of other concerns should also bring up concerns for a cardiac cause for abdominal pain. Abdominal pain, nausea and vomiting may result from hepatic congestion, rather than the more common diagnoses of gastroenteritis or appendicitis. Physical examination may reveal an enlarged liver or tender liver edge.

Initial testing should include an ECG, chest radiograph, and cardiac enzymes once heart failure is suspected [8]. A 12 lead ECG may show arrhythmias, ST changes, heart blocks, or low voltages, though the most common rhythm is sinus tachycardia [9]. A chest radiograph may reveal cardiomegaly or signs of pulmonary edema but in some cases may be completely normal. Elevations in cardiac enzymes are often seen and can help delineate the extent of disease and prognosis.

## Conclusion

Ultimately, diagnosing heart failure in the pediatric population begins with maintaining a high index of suspicion. Vital signs should be reviewed and potential diagnoses broadly considered. Additionally, physical exam should be thorough and directed. Only after the diagnosis is considered can additional testing help corroborate your diagnosis and lead the appropriate treatment. It is often difficult to make the diagnosis of heart failure if failure is not consciously considered as a possibility. Armed with knowledge and clinical suspicion, the astute physician will make this diagnosis hard to miss.

## Consent

Verbal informed consent was obtained from the family of the patient presented in case #2. Written informed consent could not be obtained from the patient in case #1 because the family was lost to follow-up. Despite repeated attempts we were unable to trace the patient or family. We believe this case report holds a worthwhile clinical lesson which could not be communicated effectively in any other way. Every effort has been made to keep the patients' identities anonymous. We would not expect the patients or their families to object to publication.

## Competing interests

The authors declare that they have no competing interests.

## Authors' contributions

DC and EK identified the cases. DC wrote up the cases and discussion. DC, EK, and JB all provided major contributions in writing and editing the manuscript. All authors read and approved the final manuscript. The authors are fully responsible for the manuscript.

## References

[B1] Wang CY Li Lu F Wu MH Lee CY Huang LM Fatal coxsackievirus A16 infection Pediatr Infect Dis J 2004 23 275 276 10.1097/01.inf.0000115950.63906.78 15014311

[B2] Bowles NE Ni J Kearney DL Pauschinger M Schultheiss HP McCarthy R Hare J Bricker JT Bowles KR Towbin JA Detection of viruses in myocardial tissues by polymerase chain reaction. evidence of adenovirus as a common cause of myocarditis in children and adults J Am Coll Cardiol 2003 42 466 472 10.1016/S0735-1097(03)00648-X 12906974

[B3] Calabrese F Rigo E Milanesi O Boffa GM Angelini A Valente M Thiene G Molecular diagnosis of myocarditis and dilated cardiomyopathy in children: Clinicopathologic features and prognostic implications Diagn Mol Pathol 2002 11 212 221 10.1097/00019606-200212000-00004 12459637

[B4] Daubeney PE Nugent AW Chondros P Carlin JB Colan SD Cheung M Davis AM Chow CW Weintraub RG Clinical features and outcomes of childhood dilated cardiomyopathy: Results from a national population-based study Circulation 2006 114 2671 2678 10.1161/CIRCULATIONAHA.106.635128 17116768

[B5] Towbin JA Lowe AM Colan SD Sleeper LA Orav EJ Clunie S Messere J Cox GF Lurie PR Hsu D Canter C Wilkinson JD Lipshultz SE Incidence, causes, and outcomes of dilated cardiomyopathy in children JAMA 2006 296 1867 1876 10.1001/jama.296.15.1867 17047217

[B6] Allegretti PJ Leonard JP Bzdusek JS A primary respiratory vs cardiac problem in a child-pediatric myocarditis Am J Emerg Med 2006 24 360 362 10.1016/j.ajem.2005.10.022 16635716

[B7] Chang YJ Chao HC Hsia SH Yan DC Myocarditis presenting as gastritis in children Pediatr Emerg Care 2006 22 439 440 10.1097/01.pec.0000221346.64991.e7 16801847

[B8] Freedman SB Haladyn JK Floh A Kirsh JA Taylor G Thull-Freedman J Pediatric myocarditis: Emergency department clinical findings and diagnostic evaluation Pediatrics 2007 120 1278 1285 10.1542/peds.2007-1073 18055677

[B9] Nakagawa M Sato A Okagawa H Kondo M Okuno M Takamatsu T Detection and evaluation of asymptomatic myocarditis in schoolchildren: Report of four cases Chest 1999 116 340 345 10.1378/chest.116.2.340 10453860

